# Functional non‐coding RNAs in vascular diseases

**DOI:** 10.1111/febs.15678

**Published:** 2021-01-07

**Authors:** Koh Ono, Takahiro Horie, Osamu Baba, Masahiro Kimura, Shuhei Tsuji, Randolph Ruiz Rodriguez, Sawa Miyagawa, Takeshi Kimura

**Affiliations:** ^1^ Department of Cardiovascular Medicine Graduate School of Medicine Kyoto University Japan

**Keywords:** atherosclerosis, circRNA, endothelial cells, lncRNA, miRNA, vascular smooth muscle cells

## Abstract

Recently, advances in genomic technology such as RNA sequencing and genome‐wide profiling have enabled the identification of considerable numbers of non‐coding RNAs (ncRNAs). MicroRNAs have been studied for decades, leading to the identification of those with disease‐causing and/or protective effects in vascular disease. Although other ncRNAs such as long ncRNAs have not been fully described yet, recent studies have indicated their important functions in the development of vascular diseases. Here, we summarize the current understanding of the mechanisms and functions of ncRNAs, focusing on microRNAs, circular RNAs and long ncRNAs in vascular diseases.

## Graphical abstract

Considerable numbers of non‐coding RNAs have been identified by RNA sequencing and genome‐wide profiling. These include microRNAs studied for decades and other non‐coding RNAs (ncRNAs) such as long ncRNAs. Recent studies have indicated their important functions in the development of vascular diseases. Here, we summarize the current understanding of the mechanisms and functions of these ncRNAs.

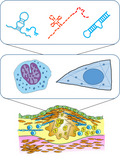

**Abbreviations**
ABCA1: ATP‐binding cassette transporter subfamily A member 1
CAD: coronary artery disease
CCL2: C‐C motif chemokine ligand 2
ChIRP: chromatin isolation by RNA purification
ceRNA: competing endogenous RNA
CHART: capture hybridization analysis of RNA targets
CLASH: cross‐linking, ligation and sequencing of hybrids
CLIP‐seq: cross‐linking immunoprecipitation sequencing
CRISPR: clustered regularly interspaced short palindromic repeat
CTGF: connective tissue growth factor
ENCODE: Encyclopedia of DNA Elements
EC: enhancer cell
eNOS: endothelial nitric oxide synthase
EZH2: enhancer of zeste homolog 2
FANTOM: Functional Annotation of the Mammalian Genome
FISH: fluorescence *in situ* hybridization
GENCODE: Encyclopedia of Genes and Gene Variants
HDL: high‐density lipoprotein
LOXL2: lysyl oxidase homolog 2
LXR: liver X receptors
MDM2: murine double minute 2
MI: myocardial infarction
PPAR: peroxisome proliferator‐activated receptor
RAP: RNA antisense purification
RIP: RNA immunoprecipitation
SHAPE: selective 2'‐hydroxyl acylation and primer extension
SPAAR: small regulatory polypeptide of amino acid response
SREBF: sterol regulatory element binding factor
VCAM1: vascular cell adhesion molecule 1
VSMC: vascular smooth muscle cell

## Introduction

Although only approximately 2% of the human genome encodes mRNAs, it is well known that a large portion of the human genome (approximately 70%) is transcribed and that the majority of the transcripts are non‐coding RNAs (ncRNAs) (Encyclopedia of DNA Elements – ENCODE, https://www.encodeproject.org; Encyclopedia of Genes and Gene Variants – GENCODE, https://www.gencodegenes.org; or Functional Annotation of the Mammalian Genome – FANTOM, https://fantom.gsc.riken.jp). The group of ncRNAs can be divided into small ncRNAs [e.g. microRNAs (miRNAs)] and transcripts > 200 nucleotides, named long ncRNAs (lncRNAs). Among the 228 000 known transcripts, approximately 7500 are classified as small ncRNAs and 48 000 are grouped as lncRNAs (GENCODE, version 34).

miRNAs constitute the vast majority of the studied group of ncRNAs. Extensive research has led to strategies to target disease‐relevant miRNAs as potential therapies. On the other hand, it has already been reported that almost 20 000 lncRNAs are functional in humans (FANTOM5) [1]. Thus, many researchers are now trying to identify lncRNAs that are related to vascular biology and diseases. However, lncRNAs have a wide variety of functions, and even their classification has not yet been fully determined.

**Fig. 1:**
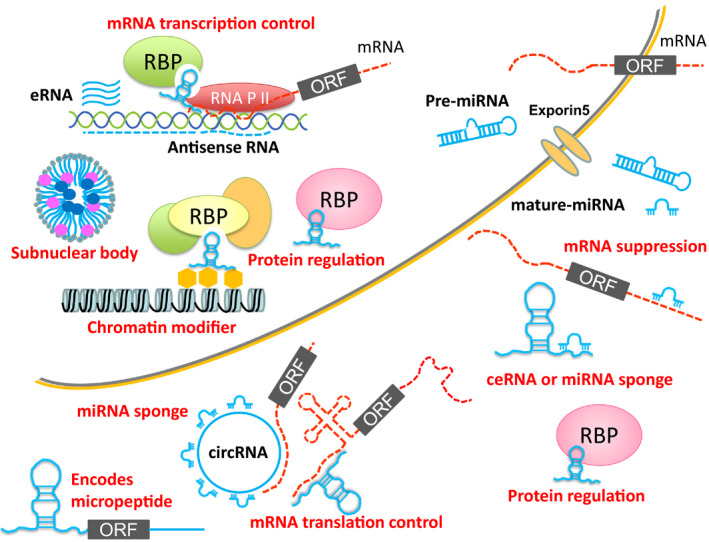
Schematic classification of ncRNAs based on the function of each ncRNA. MicroRNAs are known to suppress target mRNA functions by degradation or inhibition of translation. Some circRNAs and lncRNAs work as miRNA sponges or ceRNAs to inhibit the functions of miRNAs. lncRNAs can enhance mRNA transcription working as enhancer RNAs or by recruiting transcription factor complexes. Moreover, they can suppress mRNA transcription as antisense RNAs. Some ncRNAs may encode micropeptides at the same time. Chromatin structure can also be modified by lncRNAs. Because lncRNAs can bind to nucleic acids and proteins, there may be many other functions that are not illustrated. eRNA, enhancer RNA; RBP, RNA binding protein; RNA P II, RNA polymerase II.

In this review, we describe the current understanding and functions of miRNAs and lncRNAs, which include circular RNAs (circRNAs), in vascular biology and diseases. In particular, we try to classify lncRNAs in terms of their molecular functions and describe the current understanding and experimental methods of ncRNA research in vascular diseases (Fig. 1).

## miRNAs

miRNAs are the best studied family of ncRNAs. miRNAs are 20–25 nucleotides in length and are known to regulate protein‐coding genes through translational repression or degradation of mRNAs by binding to sequences in the 3'‐UTR of specific mRNAs. The mechanisms and the functions of miRNA are well known and strategies to target miRNAs have already been developed, including miRNA mimics to augment their functions, miRNA inhibitors (antisense against miRNA) and miRNA sponges to suppress their functions. miRNAs are stable in plasma and they have been proposed as biomarkers of myocardial infarction (MI) and other vascular diseases.

There is already extensive evidence demonstrating that miRNAs are involved in many pathological processes in vascular diseases and atherosclerosis. Recent reviews have already provided insights into the mechanisms of how miRNAs exert an influence on atherosclerosis, their potential use in diagnostics and strategies for improving RNA therapeutics [2, 3, 4]. Thus, we briefly summarize their regulation in lipid handling, inflammation and cellular mechanisms, which are involved in endothelial cell (EC) and vascular smooth muscle cell (VSMC) proliferation, migration and phenotypic switching.

### Functions of specific miRNAs in vascular diseases

Several studies have demonstrated an important role for miR‐21 with respect to negatively regulating inflammation and suppressing pro‐inflammatory signaling cascades [5, 6]. In addition to its role in regulating pro‐inflammatory responses, many studies have already reported that miR‐21 has essential functions in ECs and VSMCs [7, 8, 9]. miR‐21 targets peroxisome proliferator‐activated receptor‐α (PPAR‐α), which modulates flow‐induced endothelial inflammation. The role of miR‐21 with respect to VSMC functions and vascular remodeling is well established [10, 11]. The expression of miR‐21 is increased following balloon angioplasty or vascular injury induced by carotid artery ligation, as well as in human atherosclerotic lesions. [10, 12]. Moreover, miR­ 21 in hematopoietic cells is important for the progression of atherosclerosis. The lack of miR‐21 in hematopoietic cells enhances atherosclerosis formation [13]. In addition to its effects on vascular biology, miR‐21 has been implicated in the pathogenesis of myocardial fibrosis and hypertrophy. Currently, the investigation of oligonucleotide‐based therapeutics against miR‐21 to prevent cardiac fibrosis is underway [14].

Recent studies have indicated that miR‐33a and miR‐33b control lipid metabolism *in vivo* [15, 16, 17, 18, 19]. These miRNAs are encoded in the introns of sterol regulatory element binding factor (SREBF)2 and SREBF1, respectively, in larger mammals, including humans [16, 20]. However, there is a deletion in part of miR‐33b in rodents and miR‐33b cannot be expressed. Several groups, including our own, have reported that Abca1 and Abcg1 are the targets of miR‐33a *in vivo* through the use of either antisense oligonucleotides or by generating miR‐33a‐deficient mice [15, 16, 17, 18]. ATP‐binding cassette transporter subfamily A member 1 (ABCA1) promotes cholesterol efflux to lipid‐poor apolipoprotein A‐I and forms nascent high‐density lipoprotein (HDL) particles in the liver and intestine, whereas ABCG1 transports cellular cholesterol to HDL2 and HDL3. Therefore, upregulation of ABCA1 and ABCG1 results in a 35–50% increase in plasma HDL without affecting other lipoproteins in mice treated with anti‐miR‐33a‐oligonucleotides [15, 16, 17]. Similarly, miR‐33a‐deficient mice demonstrated a 25–40% increase in HDL [18] and showed reduced atherosclerosis formation in an *Apoe*‐deficient background [21]. On the other hand, miR‐33b knock‐in mice, in which miR‐33b is inserted in the same intron as in humans, have levels of HDL‐cholesterol that are reduced by almost 35%, in addition to severe atherosclerosis, when they are crossed with *Apoe*‐deficient mice [22, 23]. Moreover, miR‐33a deficiency ameliorates aortic aneurysm both in Ca^2+^‐ and angiotensin II‐induced mice models, which suggested that inhibition of miR‐33 may be a novel therapeutic strategy for abdominal aortic aneurysm [24]. There are several articles summarizing the role of miRNAs in lipid and lipoprotein metabolism [25, 26].

The miR‐17‐92 cluster is an important regulator of angiogenesis. It consists of six mature miRNAs including miR‐17, ‐18a, ‐19a, ‐19b, ‐20a and ‐92a, which are transcribed from a polycistronic transcription unit C13orf25 [27]. This region is closely related to the transcription factor c‐Myc, and some of these miRNAs promote angiogenesis in response to Myc. In particular, miR‐18 and miR‐19 target thrombospondin‐1 and connective tissue growth factor to promote tumor angiogenesis via the stimulation of Myc [28]. On the other hand, miR‐92a in the same cluster inhibits angiogenesis [29]. Overexpression of miR‐92a in ECs blocks the growth of new blood vessels, and inhibition of miR‐92a leads to enhanced angiogenesis and shows functional recovery from limb ischemia and MI in mice. Thus, miR‐92a may serve as an important target for the treatment of ischemic disease. The miR‐17‐92 cluster also has effects on neurological disorders [30].

miR‐126 is an endothelial‐specific miRNA encoded in intron 7 of epidermal growth factor‐like domain multiple 7 (Egfl7). Mechanosensitive transcription factor Krüppel‐like factor 2a induces miR‐126 expression for the activation of vascular endothelial growth factor signaling [31]. Thus, miR‐126 facilitates the integration of physiological stimuli with growth factor signaling in ECs to promote angiogenesis. miR‐126 also has anti‐inflammatory effects. Indeed, transfection of ECs with an oligonucleotide that decreases miR‐126 levels results in an increase in tumor necrosis factor‐α‐stimulated vascular cell adhesion molecule 1 (VCAM1) expression and increased leukocyte adherence to ECs [32]. Thus, miR‐126 overexpression can be utilized as a therapeutic approach and miR‐126‐conjugated stents have been developed to inhibit neointimal hyperplasia in rabbits. [33]. Bubble liposome‐mediated systemic delivery of miR‐126 also improved blood flow in a hind‐limb ischemia model [34].

The miR‐143/‐145 encoding genes are located in close proximity to each other on murine chromosome 18 and human chromosome 5. miR‐145 is essential for VSMC differentiation. It has been shown that miR‐145 is necessary and sufficient for directing the fate of VSMCs from multipotent neural crest stem cells [35]. miR‐145 is selectively expressed in VSMCs of the vascular wall in adult rats, and it is downregulated during the formation of neointimal lesions [36]. The target of miR‐145 is KLF5, and a corresponding increase in myocardin expression is observed by the induction of miR‐145. Overexpression of miR‐145 suppressed neointimal formation in balloon‐injured arteries and might be utilized for the treatment of vascular diseases. The lncRNA miR143HG is located in a similar locus to miR‐143/145, and it was recently implicated in cardiac specification and smooth muscle differentiation [37, 38].

miR‐221 and miR‐222 expression levels are elevated in rat carotid arteries after balloon injury [10, 39]. p27 (Kip1) and p57 (Kip2) are the target genes of miR‐221‐ and miR‐222, which mediate the effects on VSMC growth. Thus, knockdown of miR‐221 and miR‐222 results in decreased VSMC proliferation both *in vitro* and *in vivo*. Moreover, endothelial progenitor cells, quiescent ECs and umbilical vein ECs highly express miR‐221/222, which suggests an essential role for this miRNA cluster in endothelial physiology [40].

These examples only highlight some of the miRNAs related to vascular diseases (Table 1). There are many miRNAs that have similar actions and a single miRNA can have a variety of functions. Thus, any targeting therapy against miRNAs requires a specificity of action to reduce off‐target problems [41].

**Table 1:** Examples of miRNAs that have functions in vascular diseases.

miRNA	Target genes that are important for vascular diseases	Disease	References
miR‐21	PPARα, PTEN, SPRY1, SMAD7, BCL‐2	Inflammation resolution, atherosclerosis inhibition, fibrosis progression	[5, 6, 7, 8, 9, 10, 11, 12, 13, 14]
miR‐33a/b	ABCA1	Atherosclerosis inhibition, aortic aneurysm inhibition	[15, 16, 17, 18, 19, 20, 21, 22, 23, 24, 25, 26]
miR‐18 (miR‐17‐92 cluster)	CTGF	Angiogenesis promotion	[28]
miR‐19 (miR‐17‐92 cluster)	TSP1, PPARα, PTEN	Angiogenesis promotion	[28]
miR‐92a (miR‐17‐92 cluster)	ITGA5	Angiogenesis inhibition	[29]
miR‐126	SPRED1, PIK3R2, VCAM1, and ALCAM1	Angiogenesis promotion	[31, 32, 33, 34]
miR‐143	ELK1	VSMC differentiation	[35]
miR‐145	MYOCD, KLF5	VSMC differentiation	[35, 36]
miR‐221/222	KIP1, KIP2	VSMC growth	[37]

## lncRNAs

Recent work has shown that many lncRNAs are functional and represent a large class of potential therapeutic targets and agents [42]. Although clear functional classification of lncRNAs is not established yet, we will attempt to classify vascular disease‐related lncRNAs according to their molecular functions, such as genomic scaffolds, enhancers, miRNA sponges, regulators of proteins and so on. However, some lncRNAs have been shown to exhibit several different mechanisms of action concurrently. Thus, a better classification may be created in the future.

### circRNAs

In 2012, the ubiquitous expression of circRNA from genes traditionally assumed to express only mRNAs or lncRNAs was found and reported [43]. These molecules derive from a noncanonical type of splicing defined as tail‐to‐head because it goes from a downstream 5′ splice site to an upstream 3′ splice site, a process suggested to be guided by specific repetitive sequences [44]. circRNAs are enriched in the brain and it has been shown that cerebellar degeneration‐related protein 1 antisense RNA has over 70 binding sites for miR‐7, acting as a sponge for this miRNA and being able to modulate its activity on miR‐7 target genes. Several studies have addressed circRNAs expression in the cardiovascular system. The circRNA derived from alternative splicing of lipoprotein receptor 6 is named *circ_Lrp6*. It has a role as an miR‐145 sponge. Silencing of *circ_Lrp6* increases the levels of miR‐145 and thereby reduces the expression of miR‐145 target genes, such as integrin‐β8, fascin actin‐bundling protein 1, KLF‐4, YES proto‐oncogene 1 (*Yes1*) and lysyl oxidase (*Lox*). Short hairpin RNA against *circ_Lrp6* reduces the neointima formation in a model of stenosis induced by perivascular carotid collar placement in ApoE^−/−^ mice [45].

Exons of the lncRNA anti‐sense ncRNA in the *INK4* locus (*ANRIL*), which is described below, can form *circANRIL*. *circANRIL* binds to pescadillo homologue 1, an essential 60S‐preribosomal assembly factor, thereby impairing exonuclease‐mediated pre‐ribosomal RNA processing and ribosome biogenesis in VSMCs and macrophages. As a consequence, *circANRIL* induces nucleolar stress and p53 activation, resulting in the induction of apoptosis and inhibition of proliferation, which are key cell functions in atherosclerosis [46].

The Nrg‐1‐ICD‐induced circular alpha‐actin‐2 (*circACTA2*) acts as a sponge, which binds miR‐548f‐5p. *circACTA2* upregulates α‐smmoth muscle actin expression by the suppression of miR‐548f‐5p, thereby facilitating stress fiber formation and cell contraction in human arterial smooth muscle cells [47].

### lncRNAs that modulate chromatin architecture

Many lncRNAs are known to affect the transcription of genes. They often impact chromatin readers and writers to control the gene promoter [48]. Mechanistically, lncRNAs can recruit chromatin remodelers to target genes via binding to DNA, or control their enzymatic activity or function as negative decoys. Such lncRNAs with relevance to atherosclerosis include *ANRIL*, *lincRNA‐p21*, *MALAT1*, *MEG3*, *TUG1*, *GAS5* and *MANTIS*.

*ANRIL* is transcribed from the well‐known 9p21.3 cardiovascular disease locus, which has been strongly implicated in coronary artery disease (CAD) through genome‐wide association studies [49, 50, 51, 52]. Although important tumor suppressor genes *CDK2A* and *CDKN2B* are found near this region, these genes are not dysregulated in animal models of atherosclerosis or human samples. Indeed, the approximately 60‐kb risk haplotype is human‐specific and lacks coding genes, hindering efforts to clarify its function. Recently, induced pluripotent stem cells from risk and non‐risk individuals were generated. After differentiation into VSMCs, risk VSMCs exhibit globally altered transcriptional networks that resemble the previously identified CAD risk genetic pathways. Of note, deleting the risk haplotype rescues VSMCs, whereas expressing *ANRIL* induces cardiovascular risk phenotypes in non‐risk VSMCs [53]. Some part of the functions of *ANRIL* is mediated by interactions among *ANRIL*, enhancer of zeste homolog 2 (EZH2; as part of the polycomb repressive complex 2 complex) and the histone acetyltransferase p300 [54].

*lincRNA‐p21* is downregulated in a mice model of atherosclerosis and in patients with CAD. Silencing of *lincRNA‐p21* was shown to induce cell proliferation and inhibit apoptosis in VSMCs and macrophages *in vitro*. Moreover, inhibition of *lincRNA‐p21* increased neointimal hyperplasia in a carotid artery injury model *in vivo*. Mechanistically, *lincRNA‐p21* enhances p53 transcriptional activity via binding to the E3 ubiquitin ligase mouse double minute 2 (MDM2). The association of *lincRNA‐p21* and MDM2 releases MDM2 repression of p53, thereby enabling p53 to interact with p300, which enhances p53 transcriptional activity [55].

Metastasis‐associated lung adenocarcinoma transcript 1 (*MALAT1*) was originally identified as a nuclear‐enriched prognostic lung cancer metastasis marker. Genetic deletion or silencing of *MALAT1* *in vivo* inhibits EC proliferation, postnatal retina vascularization and ischemia‐induced neovascularization [56]. In humans, reduced *MALAT1* expression levels are associated with a worse prognosis [57]. A recent study suggests that the action of *MALAT1* is mediated by interaction with polycomb repressive complex 2, binding of the transactivation domain of TEAD proteins, activities of competing endogenous RNAs (ceRNAs) and the regulation of various signaling pathways, including phosphatidylinositol‐3‐kinase‐AKT, mitogen‐activated protein kinase, WNT and nuclear factor‐kappa B [58].

Maternally expressed gene 3 (*MEG3*) was previously shown to regulate tumor suppressor genes through stimulating p53 accumulation [59]. GapmeR‐mediated silencing of *MEG3* in aged mice promotes neovascularization after hindlimb ischemia *in vivo* [60]. In humans, *MEG3* is significantly downregulated in the lung tissue of patients with pulmonary arterial hypertension [61]. Mechanistically, two distal motifs interact by base pairing to form alternative, mutually exclusive pseudoknot structures, which are called ‘kissing loops’, in an evolutionarily conserved region of *MEG3*. Mutations that destroy these interactions impair *MEG3*‐dependent p53 stimulation *in vivo* [62]. In addition to the *MEG3*‐p53 interaction, several studies have suggested that it functions as a sponge for miR‐9, ‐21, ‐26 and ‐328 [63, 64, 65, 66].

Taurine up‐regulated gene 1 (*TUG1*) is expressed in rat VSMCs and its level is increased in synthetic VSMCs [67]. Because EZH2‐mediated methylation of α‐actin is dependent on *TUG1*, F‐actin polymerization is promoted by *TUG1* in synthetic VSMCs.

Growth arrest‐specific transcript 5 (*GAS5*), which is located antisense to another lncRNA, *GAS5 antisense*, was identified and named as a result of its elevation upon cell growth arrest. Overexpressed *GAS5* increases lipid accumulation in THP‐1 macrophages. *GAS5* inhibits the expression of ABCA1 by binding to EZH2. Knockdown of *GAS5* promotes reverse‐transportation of cholesterol and inhibits intracellular lipid accumulation, ultimately preventing atherosclerosis progression [68]. *GAS5* was also shown to have multiple molecular mechanisms, such as binding to DNA sequences and forming an RNA–DNA triplex complex, which results in the triggering or suppression of the expression of genes in human cancer [69].

Recently, epigenetically controlled lncRNAs in human umbilical vein ECs were searched using an exon‐array technique after silencing histone demethylase JARID1B. *MANTIS* was identified as the most strongly regulated lncRNA [70]. Deletion or silencing of *MANTIS* inhibits angiogenic sprouting and alignment of ECs in response to shear stress. Mechanistically, the nuclear *MANTIS* interacts with BRG1, a subunit of the switch/sucrose nonfermentable (SWI/SNF) chromatin remodeling complex. This interaction is required for nucleosome remodeling and the regulation of key endothelial genes such as SMAD6, SOX18 and COUP‐TFII by facilitating the recruitment of RNA polymerase II to their promoter regions.

### lncRNAs work as enhancer RNAs or regulate neighboring mRNAs

Many ncRNAs were found to be transcribed as active enhancers for gene promoters and they are called enhancer lncRNAs, which confer enhancer activity by capturing the promoter‐contacting mediator protein complex. There are also ncRNAs that act in *cis* to regulate their neighboring mRNAs. Examples of such important ncRNAs in the field of atherosclerosis include *HOTTIP*, *LEENE*, *SMILR* and *lncRNA‐CCL2*.

HOXA transcript at the distal tip (*HOTTIP*) expression level is higher in CAD tissues than in normal arterial tissues. Ectopic expression of *HOTTIP* promotes EC proliferation and increases the expression of cyclin D1 and PCNA. On the other hand, downregulated expression of *HOTTIP* suppresses EC proliferation and migration [71].

lncRNA that enhances endothelial nitric oxide synthase (eNOS) expression (*LEENE*) was identified by combining RNA‐sequencing (RNA‐seq) techniques and chromatin conformation capture methods [72]. *LEENE* facilitates the recruitment of RNA Pol II to the eNOS promoter region to enhance eNOS mRNA transcription.

Smooth muscle‐induced lncRNA enhances replication (*SMILR*) was identified as an lncRNA for which the expression was altered in human saphenous vein VSMCs following stimulation with interleukin‐1α and platelet‐derived growth factor. Mechanistically, the expression of genes proximal to *SMILR* was also altered by treatment with these cytokines. In addition, *HAS2,* which is one of the proximal transcripts of *SMILR*, was also reduced by *SMILR* knockdown. Increased expression of *SMILR* is observed in unstable atherosclerotic plaques and in plasma from patients with high plasma C‐reactive protein [73].

*lncRNA‐CCL2* is transcribed divergently to C‐C motif chemokine ligand 2 (CCL2), a pro‐atherosclerotic chemokine. *lncRNA‐CCL2* and CCL2 are up‐regulated in response to inflammatory stimuli, and their expression is elevated in unstable human atherosclerotic plaques [74]. Knockdown experiments showed the positive regulation of CCL2 by lncRNA‐CCL2. This regulation involves the interaction of *lncRNA‐CCL2* with RNA binding proteins such as HNRNPU and IGF2BP2.

### lncRNA localized in the subnuclear body

Some lncRNAs can affect other genes through their architectural roles in subnuclear territories. A previous study reported that the nuclear enriched abundant transcript 1 (*NEAT1*) is critical for the structural constituent of paraspeckles and tumorigenesis by promoting cell migration and proliferation [75]. Moreover, another study provided evidence demonstrating a critical role for *NEAT1* with respect to promoting VSMC proliferation, migration and dedifferentiation during phenotypic switching. A loss‐of‐function study of *NEAT1* in VSMCs resulted in enhanced expression of smooth muscle‐specific genes at the same time as attenuated VSMC proliferation and migration [76]. Mechanistically, *NEAT1* sequesters the key chromatin modifier WD Repeat Domain 5 (WDR5) from smooth muscle‐specific gene loci and initiates an epigenetic off state, which consequently impairs SRF accessibility to the CArG boxes, resulting in down‐regulation of smooth muscle‐contractile gene expression.

### lncRNAs that work as ceRNAs to absorb miRNAs

There are several lncRNAs that work as ceRNAs to absorb miRNAs. However, most of them are not sufficiently highly expressed compared to the number of corresponding target miRNAs per cell.

*H19* is among the first discovered eukaryotic lncRNAs and is known to be transcribed as intergenic RNA from the imprinted *H19/IGF2* gene locus [77]. Although it is downregulated after birth, some vascular diseases are accompanied by re‐expression of this lncRNA. Increased expression of H19 is reported in aortic aneurysms and in calcific aortic valves [78, 79, 80]. Because of a high degree of secondary structure conservation, H19 is assumed to function as a structure‐dependent lncRNA. However, miR‐675‐3p and ‐5p are also encoded in H19, which may play a role in disease progression [81]. Moreover, H19 has a potential binding site for the let‐7 miRNA family and may also work as a molecular sponge [82].

Cholesterol homeostasis regulator of miRNA expression (*CHROME*) was identified as an important regulator of cellular and systemic cholesterol homeostasis [83]. *CHROME* levels are elevated in the plasma and atherosclerotic plaques of patients with CAD. It has been shown that *CHROME* promotes cholesterol efflux and HDL biogenesis by changing the levels of miRNAs that repress genes in these pathways. Indeed, *CHROME* binds specifically to miR‐27b, miR‐33a, miR‐33b and miR‐128, which are miRNAs that repress genes mediating cholesterol transport.

Through a genome‐wide association study using single nucleotide polymorphisms, chromosome 22q12.1 was identified as a susceptible locus for MI. Within this locus, a novel ncRNA was isolated and designated as MI‐associated transcript (*MIAT*). *MIAT* consists of five exons and does not encode any translational product. Several studies have reported that *MIAT* functions as a sponge for many miRNAs to regulate tumorigenesis and progression. These include miRNA‐155‐5p, miR‐29c, miR‐141 and miR‐212. *MIAT* also functions as a ceRNA against miR‐150‐5p to form a feedback loop with vascular endothelial growth factor [84]. Moreover, *MIAT* also acts to sponge miR‐204‐5p in the *MIAT*/miR‐204‐5p/HMGB1 axis in cerebral ischemia [85].

### lncRNAs that bind to and regulate proteins

Liver X receptors (LXRs) are transcription factors that regulate cellular and systemic cholesterol homeostasis. Liver‐expressed LXR‐induced sequence (*LeXis*) was shown to control LXRs [86]. Hepatic *LeXis* expression is strongly induced in response to a Western diet or pharmacological LXR activation. Changes in *LeXis* levels in the liver affect the levels of cholesterol biosynthesis‐related genes. This effect is mediated by the interaction of *LeXis* with the heterogeneous ribonucleoprotein RALY, which acts as a transcriptional cofactor for cholesterol biosynthetic genes in the mouse liver.

Macrophage‐expressed LXR‐induced sequence (*MeXis*) was identified as an amplifier of LXR‐dependent transcription of Abca1, which is an important regulator of cholesterol efflux [87]. Mice lacking the *MeXis* show reduced Abca1 expression in a tissue‐selective manner. Moreover, *MeXis*‐deficient bone marrow cells altered chromosome architecture at the Abca1 locus and accelerated the development of atherosclerosis in mice. Mechanistically, *MeXis* interacts with and guides transcriptional coactivator DDX17 to the promoter region of Abca1 in a context‐specific manner.

### lncRNAs that are transcribed as antisense RNAs

Some lncRNAs reside within protein‐coding gene units and overlap coding exons in antisense. Because the effects on host genes can be positive, negative or neutral, this classification is not directly related to their functions. Examples of such ncRNAs include *ANRIL*, *MALAT1*, *HOXC‐AS1*, *SENCR*, *GATA6‐AS*, *STEEL* and NEXN‐AS1. The functions of *ANRIL* and *MALAT1* have already been described above.

HOXC cluster antisense RNA 1 (*HOXC‐AS1*) and homeobox C6 (*HOXC6*) were shown to be downregulated in carotid atherosclerosis via microarray analysis. Lentivirus‐mediated overexpression of *HOXC‐AS1* induces *HOXC6* expression at mRNA and protein levels in THP‐1 macrophages [88]. However, the precise functions of *HOXC‐AS1* in atherosclerosis need to be clarified in further experiments.

Smooth muscle and EC‐enriched migration/differentiation‐associated lncRNA (*SENCR*) was revealed by RNAseq) of human coronary artery SMCs. *SENCR* is transcribed antisense from the 5'‐end of the *FLI1* gene and two splice variants exist [89]. Knockdown studies revealed little to no *cis*‐acting effect of *SENCR* on FLI1 or neighboring gene expression. Loss‐of‐function studies indicated that *SENCR* inhibits SMC migration and maintains EC membrane integrity. Mechanistically, *SENCR* physically associates with cytoskeleton‐associated protein 4, thereby stabilizing cell membrane‐bound cadherin‐5 to promote EC adherens junction integrity [90].

GATA transcription factors are involved in variety of processes in development and diseases. The GATA locus expresses a noncoding antisense transcript of GATA6, named *GATA6‐AS* [91]. *GATA6‐AS* is upregulated in ECs during hypoxia. Silencing of GATA6‐AS diminished transforming growth factor‐β2‐induced endothelial–mesenchymal transition and promoted blood vessel formation in mice. Lysyl oxidase homolog 2 (LOXL2), which is known to remove activating H3K4me3 chromatin marks, is identified as a direct binding partner of *GATA6‐AS*. Moreover, a set of angiogenesis‐related genes were inversely regulated by LOXL2 and *GATA6‐AS* negatively regulated nuclear LOXL2 function. Thus, GATA6‐AS controls EC function as a negative regulator of nuclear LOXL2 function and activates angiogenesis‐related genes by increasing H3K4me3 methylation.

Spliced‐transcript endothelial‐enriched lncRNA (*STEEL*) is expressed from the homeobox D locus and is transcribed as antisense to homeobox D transcription factors [92]. STEEL promotes blood vessel formation *in vivo*. *STEEL* up‐regulates both eNOS and KLF2 and is inhibited by both of them in a feedback manner. Mechanistically, up‐regulation of eNOS and KLF2 is mediated via the recruitment of poly‐ADP ribosylase, PARP1 by *STEEL*. Feedback inhibition of *STEEL* expression may modulate angiogenic behavior in a position‐ and shear‐dependent fashion.

Nexilin F‐actin binding protein antisense RNA 1 (*NEXN‐AS1*) interacts with the chromatin remodeler BAZ1A and upregulates the expression of the actin‐binding protein NEXN. NEXN deficiency results in enhanced atherosclerosis, whereas NEXN overexpression reduces atherosclerosis in mice model of atherosclerosis. Both NEXN‐AS1 and NEXN are reduced in human atherosclerotic plaques, and patients with CAD have lower plasma NEXN levels [93].

### lncRNAs that encode micropeptides

Emerging evidence indicates that several lncRNA molecules have short ORFs that encode functional peptides. *LINC00961* was first annotated as a lncRNA but reassigned as a protein coding gene for the small regulatory polypeptide of amino acid response (SPAAR) micropeptide. *LINC00961* was increased during the differentiation in ECs. Silencing of *LINC00961* via siRNA or a GapmeR strategy significantly reduced EC adhesion, tube formation, migration, proliferation and endothelial membrane barrier integrity. On the other hand, overexpression of the SPAAR ORF increased tubule formation. Furthermore, overexpression of an ATG mutant of the full length *LINC00961* transcript reduced network formation, which suggests that a ncRNA function of the transcript is opposed to the effects of SPAAR. Mechanistically, *LINC00961* RNA binds the G‐actin sequestering protein thymosin beta‐4 (Tβ4) and Tβ4 depletion acts similarly to the overexpression of the ATG mutant. SPAAR binding partners includes the actin binding protein, spectrin repeat containing nuclear envelope protein 1 [94].

Currently, many lncRNAs related to vascular diseases are being identified and a summary of the lncRNAs is provided in Table 2.

**Table 2:** Examples of lncRNAs that have functions in vascular disease.

lncRNA	Function	Disease	References
*circ_Lrp6*	CeRNA/miRNA sponge	Atherosclerosis progression	[45]
*CircANRIL*	Inhibiting rRNA processing	Atherosclerosis inhibition	[46]
*circACTA2*	CeRNA/miRNA sponge	VSMC differentiation	[47]
*ANRIL*	Transcription Guiding chromatin regulators	CAD progression	[53, 54]
*lincRNA‐p21*	Transcription Protein regulation	CAD progression	[55]
*MALAT1*	Transcription Binding chromatin remodelers	EC proliferation	[56, 57, 58]
*MEG3*	Tethering of chromatin modifier CeRNA/miRNA sponge	EC and VSMC proliferation inhibition Pulmonary hypertension inhibition	[59, 60, 61, 62, 63, 64, 65, 66]
*TUG1*	3D chromatin positioning	VSMC proliferation	[67]
*GAS5*	Transcription	Macrophage lipid accumulation	[68, 69]
*MANTIS*	Scaffold of chromatin modifier	EC angiogenesis promotion	[70]
*HOTTIP*	eRNA Transcription	EC proliferation	[71]
*LEENE*	eRNA Transcription	EC inflammation inhibition	[72]
*SMILR*	eRNA Transcription	VSMC proliferation Atherosclerosis promotion	[73]
*lncRNA‐CCL2*	eRNA Transcription	Macrophage chemotaxis promotion	[74]
*NEAT1*	Decoy for chromatin regulator	VSMC proliferation	[75, 76]
*H19*	CeRNA/miRNA sponge mRNA decay	Aortic aneurysm and aortic valve calcification promotion	[77, 78, 79, 80, 81, 82]
*CHROME*	CeRNA/miRNA sponge	Cholesterol efflux promotion	[83]
*MIAT*	CeRNA/miRNA sponge	EC proliferation	[84, 85]
*LeXis*	Transcription	Cholesterol biosynthesis promotion	[86]
*MeXis*	Transcription	Cholesterol efflux promotion	[87]
*HOXC‐AS1*	Transcription	Atherosclerosis inhibition	[88]
*SENCR*	Transcription	VSMC migration inhibition	[89, 90]
*GATA6‐AS*	Transcription Binding chromatin modifier	Angiogenesis inhibition	[91]
*STEEL*	Transcription	Angiogenesis promotion	[92]
*NEXN‐AS1*	Binding chromatin modifier	Atherosclerosis inhibition	[93]
*LINC00961*	Encoding micropeptide	Angiogenesis inhibition	[94]

## Strategies for the identification of functional ncRNAs

There are already many databases of published miRNA sequences, annotations and predicted targets (e.g. http://www.mirbase.org and http://www.targetscan.org/vert_72). On the other hand, genome‐wide transcriptomic approaches such as RNA‐seq, microarray and cap analysis of gene expression technology are currently being used for the detection of lncRNAs [95]. Recently, a computational approach was also applied for the identification of lncRNAs as a result of an improvement in lncRNA annotations from RNA‐seq data [1]. RNA‐seq is superior with respect to detecting low‐abundance transcripts, although arrays have the advantage of rapid analysis.

After the identification, validation of the transcripts is necessary for the next step of the analysis. Quantitative‐PCR is commonly utilized for the validation of the candidate ncRNAs expression and the RACE technique is applied for the identification of the full sequence of the lncRNA. Moreover, identification of the localization of the lncRNA is useful and important with respect to hypothesizing its potential biological functions. Precise information of its subcellular localization can be obtained by RNA fluorescence *in situ* hybridization (FISH) [96]. Determination of tissue expression is also important.

As mentioned earlier above regarding lncRNAs that encode micropeptides, it is necessary to test the coding potential of novel lncRNAs. For example, a dwarf ORF encoded by an annotated lncRNA was reported to encode a peptide of 34 amino acids with unknown function [97].

Several techniques have also been developed for the identification of the interactions between lncRNAs and the genome, RNAs and proteins. Just as chromatin immunoprecipitation followed by microarray or deep sequencing has greatly improved our understanding of protein–DNA interactions on a genomic scale, chromatin isolation by RNA purification (ChIRP) was first developed by Chu *et al*. [98] to map long RNA occupancy genome‐wide at high resolution. This method is based on the affinity of antisense DNA oligonucleotides to capture the target lncRNA:chromatin complexes, which then generates a map of genomic binding sites. Other high‐throughput experimental technologies include capture hybridization analysis of RNA targets (CHART), RNA antisense purification (RAP), RNA immunoprecipitation (RIP), cross‐linking immunoprecipitation sequencing (CLIP‐seq), ChIRP‐mass spectrometry (MS) and CHART‐MS. These techniques have led to a rapid expansion of lncRNA research and also resulted in many publicly available databases [99, 100, 101, 102, 103]. RNA–RNA interactions can also be assessed by ChIRP easily because the design of affinity‐probes is straightforward; however, it cannot differentiate direct RNA–RNA binding from possible interactions with other intermediate proteins. By contrast, cross‐linking, ligation and sequencing of hybrids (CLASH) can be utilized to detect only direct hybridization between RNA molecules [104].

The unique secondary and tertiary structure of each lncRNA may contribute to its biological function. Thus, several techniques have been developed to obtain the RNA structure. Recent advances in probing the RNA structurome, including the use of RNA‐selective 2′‐hydroxyl acylation and primer extension (SHAPE) or kethoxal reagents or dimethyl sulfate, can provide unprecedented insights into the architecture of RNA molecules in living cells [105, 106, 107]. However, it is still unclear what controls lncRNA folding in the complex nuclear environment and to what extent secondary and tertiary structures are important to mediate lncRNA function.

Finally, loss‐of‐function strategies are required to determine the physiological functions of lncRNAs in animal models. Typical knockdown assays make use of short hairpin RNAs, siRNAs or locked nucleic acid GapmeRs. Their results can be assigned with higher confidence when oligonucleotide‐based strategies are complemented by the recent development of clustered regularly interspaced short palindromic repeat (CRISPR) technology [108, 109]. A summary of lncRNA analysis is provided in Table 3.

**Table 3:** Summary of ncRNA research techniques.

Stage	Technique	References
Identification	Microarray, RNA‐seq, Cap‐assisted gene expression sequencing, and nuclear run‐on assay	[95]
Validation	Database, quantitative PCR, RACE and RNA‐FISH	[96]
Assessment of the coding potential	Bioinformatic tool, and *in vitro* transcription assays	[97]
Genome‐wide mapping of binding sites	ChIRP, Chart, RAP, RIP and CLIP‐seq	[98, 99, 100, 101]
Identification of binding proteome	ChIRP‐MS and Chart‐MS	[102, 103]
Identification of RNA‐RNA interaction	CLASH	[104]
Analysis of RNA secondary structure	SHAPE and use of kethoxal reagents or dimethyl sulfate	[105, 106, 107]
Identification of function *in vivo*	Genetic KO, promoter insertion, polyA insertion, RNA interference, CRISPR interference and transgenics	[108, 109]

## Future perspectives

**Fig. 2:**
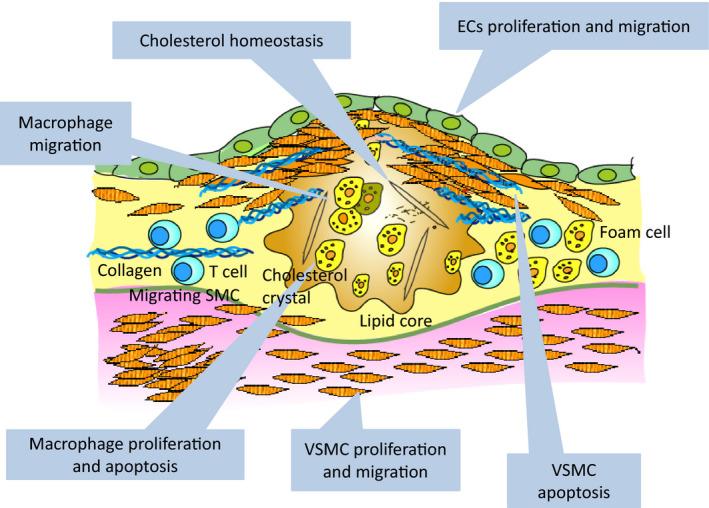
Possible targets of ncRNAs in atherosclerosis formation. EC proliferation and migration is affected by miR‐21, miR‐126, miR‐221/222, ANRIL, MIAT, MEG3, MALAT1, MANTIS, HOTTIP and LINC00961. VSMC proliferation and migration is mediated by miR‐21, miR‐143/145, H19, lncRNA‐p21, circ‐Lrp6, TUG1, SMILR, NEAT1 and SENCR. VSMC apoptosis is affected by miR‐125b, lncRNA‐p21 and circ‐ANRIL. Macrophage proliferation and apoptosis is enhanced by miR‐19 and lncRNA‐p21 and macrophage migration is mediated by lncRNA‐CCL2. Cholesterol homeostasis is also important for atherosclerosis formation, which is regulated by miR‐33a/b, GAS5, CHROME, MeXis and LeXis (liver).

In summary, recent studies have provided considerable evidence about the functions of miRNAs and lncRNAs on various stages of cardiovascular diseases. They have indicated that these ncRNAs participate especially in the regulation of a broad spectrum of atherosclerosis (Fig. 2). Most of the ncRNAs have their own promoter and are regulated by distinct transcriptional factors in the disease condition. However, some of miRNAs are located within an intron of its host gene and the regulation totally depends on the levels of their host genes.

Currently, RNA therapeutics are diverse and include antisense oligonucleotides, siRNAs, miRNAs, mRNAs, RNA aptamers, short activating RNAs and single guide RNAs for CRISPR/Cas9 systems. Molecular functions for miRNAs are relatively clear, and anti‐miRs and miRNA mimics have been developed. Anti‐miRs bind directly to the target miRNA and inhibit its function. Locked nucleic acids enhance the function of anti‐miRs by increasing their affinity and stability [110]. miRNA mimics are synthetic, double‐stranded RNAs that resemble a naturally generated miRNA. Several miRNAs that are currently being investigated in clinical trials are summarized in Table 4.

**Table 4:** miRNAs that are currently being investigated in clinical trials.

miRNA	Inhibition or augmentation	Name of drug	Target disease	Clinical stage	Year and clinical trial number
miR‐122	Inhibition	Miravirsen	Hepatitis C	Phase II	2010/2015 NCT01200420 NCT02508090 NCT02452814
miR‐103/107	Inhibition	RG‐125 (AZD4076)	Nonalcoholic steatohepatitis	Phase I	2015 NCT02612662
miR‐21	Inhibition	SAR339375	Alport’s syndrome	Phase II	2016 NCT02855268
miR‐155	Inhibition	MRG‐106 (Cobomarsen)	Cutaneous T cell lymphoma Mycosis fungoides/lymphoma and leukemia	Phase II/ Phase I	2018/2015 NCT03713320/ NCT02580552
miR‐92a	Inhibition	MRG‐110	Wound	Phase I	2018 NCT03603431
miR‐16	Augmentation	TargomiRs	Malignant pleural mesothelioma Non‐small cell lung cancer	Phase I	2015 NCT02369198
miR‐29	Augmentation	MRG‐201	Keloid	Phase II	2018 NCT03601052

On the other hand, for most of the lncRNAs, the molecular mode of action remains elusive. Thus, further investigations are required aiming to understand the functions of lncRNAs *in vivo*. In any case, inhibition or activation of lncRNAs also leads to beneficial effects on disease conditions; therefore, the development of techniques that enable the fine regulation of lncRNAs is awaited.

## Conflicts of interest

The authors declare no conflict of interest.

## Author contributions

KO prepared the manuscript. TK, OB, MK, ST, RRR, SM, and TK analyzed current data and discussed the contents.
